# Global, regional, and national burden of ischemic stroke in older adults (≥60 years) from 1990 to 2021 and projections to 2030

**DOI:** 10.3389/fneur.2025.1567609

**Published:** 2025-05-08

**Authors:** Shuting Hua, Zexian Dong, Hui Wang, Tong Liu

**Affiliations:** ^1^The Fifth Clinical College of Guangzhou University of Chinese Medicine, Guangzhou, China; ^2^School of Medicine, Jinan University, Guangzhou, China; ^3^Guangdong Provincial Second Hospital of Traditional Chinese Medicine, Guangzhou, China; ^4^Department of Acupuncture and Rehabilitation, Guangdong Provincial Second Hospital of Traditional Chinese Medicine, Guangzhou, China; ^5^Guangdong Provincial Key Laboratory of Research and Development in Traditional Chinese Medicine, Guangzhou, China

**Keywords:** ischemic stroke, elderly population, global burden, socio-demographic index, epidemiological trends, public health policy

## Abstract

**Background:**

Ischemic stroke is a leading cause of disability and mortality among adults worldwide, particularly in the older population (≥60 years). With the accelerating global aging population, it is crucial to analyze the trends and influencing factors of the global, regional, and national burden of ischemic stroke and forecast future trends. These insights are essential for informing the formulation of public health policies.

**Methods:**

Using data from the Global Burden of Disease (GBD) 2021 database, this study examined the age-standardized incidence, age-standardized prevalence, age-standardized mortality, and age-standardized disability-adjusted life years (DALYs) of ischemic stroke in individuals aged 60 years and above from 1990 to 2021. A combination of variables, including the socio-demographic index (SDI), sex, and age groups, was applied in regression analyses and Bayesian predictive models to examine trends and forecast the burden of ischemic stroke up to 2030.

**Results:**

From 1990 to 2021, despite global population growth among older adults, the age-standardized incidence, age-standardized prevalence, age-standardized mortality, and age-standardized disability-adjusted life years of ischemic stroke demonstrated an overall declining trend (all EAPCs were negative). The decline in disease burden was most pronounced in high-SDI regions, while low-SDI regions faced a significantly higher disease burden and exhibited notable regional disparities. The overall burden of ischemic stroke was higher in males than in females; however, in the 80–84 age group, females exceeded males in disease burden. Projections indicate that by 2030, the burden of ischemic stroke in older adults globally will continue to decline. Nevertheless, due to the aging population, the absolute number of patients is expected to increase.

**Conclusion:**

The global burden of ischemic stroke has significantly decreased, particularly in high-SDI regions with abundant healthcare resources. However, low-SDI regions face more substantial public health challenges. It is recommended to enhance the control of high-risk factors such as hypertension, smoking, and high BMI, and to optimize healthcare services in low-income regions to further reduce the burden of ischemic stroke and improve the quality of life for older adults.

## 1 Introduction

Stroke is one of the leading causes of adult disability and mortality globally, posing a significant threat to public health. Among the various types of stroke, ischemic stroke (IS) constitutes a substantial proportion (1). From 1990 to 2019, IS was a major contributor to disability-adjusted life years (DALYs) in individuals aged 50 and above, highlighting its severe impact on global health (2). With the accelerated aging of the global population, the risk of IS among older adults (≥60 years) continues to rise, resulting in an increasing disease burden and becoming a critical public health challenge that requires urgent attention (1–3).

Previous studies have demonstrated that the incidence, mortality, and DALYs of IS exhibit distinct distributions and trends globally (1). Within the Global Burden of Disease (GBD) database, the epidemiological metrics related to IS are defined as follows. The age-standardized incidence rate (ASIR) quantifies the frequency of newly diagnosed cases in a population over a specific period by eliminating differences in age structure, thereby reflecting the level of exposure to risk factors. The age-standardized mortality rate (ASMR), after adjusting for age confounding, not only represents the risk of death from the disease but also highlights regional disparities in healthcare resource allocation and treatment capabilities. The age-standardized prevalence rate (ASPR) effectively captures the cumulative burden of existing cases and the quality of long-term disease management. In our study, we standardize all these indicators using the WHO standard population to ensure comparability across diverse regions and time periods. Without this standardization, inherent variations in population age structures could distort the true trends in disease burden, ultimately resulting in misleading comparisons and inaccurate assessments. Additionally, the age-standardized DALY rate (ASDR) serves as a supplementary metric by integrating years of life lost due to premature death with years lived with disability, thereby providing a comprehensive temporal assessment of the disease’s impact on population health expectancy (4). In subsequent analyses, we will consistently use the abbreviations ASIR, ASMR, ASPR, and ASDR to denote these respective indicators. According to the 2019 GBD study, stroke is the second leading cause of death worldwide and the third leading contributor to increased DALYs, with IS accounting for 85% of all stroke cases (1, 2). Between 1990 and 2019, although the age-standardized incidence, mortality, and DALYs rates of IS declined globally, factors such as population growth and aging led to increases in new cases, deaths, survivors, and individuals living with disabilities due to IS. Significant regional disparities in disease burden exist, with lower SDI regions bearing heavier burdens, and sex differences also influencing the burden (1–3).

Regionally, the burden of IS varies considerably. For example, between 1990 and 2019, regions such as East Asia, Eastern Europe, Central Asia, North Africa, the Middle East, and Eastern Sub-Saharan Africa had higher burdens of IS, whereas Western Europe, Australia, New Zealand, high-income Asia Pacific, high-income North America, and Andean Latin America experienced relatively lower burdens (1, 2). These disparities likely arise from socioeconomic development, healthcare accessibility, lifestyle, and exposure to risk factors (1–3).

The socio-demographic index (SDI) is a composite metric closely associated with health outcomes, reflecting the developmental status of various countries and regions (1, 2). Research has shown that high-SDI regions generally possess better resources and capacities for preventing, diagnosing, and treating IS, leading to lower disease burdens. Conversely, low-SDI regions face higher IS burdens and greater challenges in healthcare accessibility and quality (1, 2).

Nationally, the burden of IS also varies significantly. According to GBD 2019 data, from 1990 to 2019, Egypt had the largest increase in ASIR globally, with an estimated annual percentage change (EAPC) of 1.40 (95% CI: 1.27–1.52). Similarly, China showed a notable increase, with an EAPC of 1.10 (95% CI: 1.00–1.20) (2). In contrast, despite higher overall medical standards, countries like the United States still face a substantial IS burden due to factors such as accelerated population aging (1, 2).

Sex is another important factor influencing the burden of IS. Studies have demonstrated sex differences in the incidence, mortality, and DALYs rates of IS, with these differences varying across age groups and regions (1–3). Understanding these disparities is crucial for developing targeted prevention and intervention strategies aimed at reducing the impact of IS on different populations.

Previous research has identified major global risk factors for IS, including hypertension, smoking, high body mass index (BMI), elevated blood glucose, and hyperlipidemia. Despite extensive discussions on the burden of IS, research focusing specifically on older adults (≥60 years) remains limited, particularly in forecasting future disease burdens. Given that the risk factors for IS are highly preventable (5, 6) and that stroke epidemiology is rapidly evolving, regular updates to the GBD framework are necessary (2, 3). Studies have confirmed that the incidence of IS is significantly higher among older adults compared to younger populations (2). Moreover, as global population aging intensifies, older adults with IS face poorer prognoses and heavier disease burdens compared to younger patients (1, 2).

In summary, this study addresses a notable gap in the existing literature, which predominantly focuses on the disease burden across all age groups while paying insufficient attention to the unique pathophysiological characteristics of the elderly (endothelial dysfunction, cerebral arteriosclerosis, etc.) and the consequent amplification of IS burden in this population. Utilizing the latest GBD 2021 data system, we have innovatively developed a time-dimension decomposition model to systematically analyze the spatiotemporal evolution and geographical distribution of IS burden among individuals aged 60 and above worldwide from 1990 to 2021. We hypothesize that, in the context of accelerated population aging, the global burden of IS in the elderly will continue to intensify, with regions of lower SDI experiencing relatively greater growth pressure. By elucidating the uneven distribution of disease burden and its underlying driving factors, our study provides critical evidence for optimizing the regional allocation of public health resources, particularly in the formulation of targeted IS prevention and control strategies for the elderly. Ultimately, this research not only offers a valuable theoretical supplement to the understanding of global trends in IS among the elderly but also establishes a robust theoretical and data-driven foundation for future research, policy-making, and practical interventions aimed at reducing the disease burden, improving quality of life, and decreasing disability and mortality in this vulnerable population.

## 2 Methods

### 2.1 Data sources

The data for this study were exclusively obtained from the GBD 2021 database.^1^ This database contains burden data for 371 diseases and 88 risk factors, encompassing 204 countries and regions globally, and utilizes a standardized methodology for data collection and indicator evaluation. The study specifically extracted data on ischemic stroke (IS) in individuals aged 60 years and older from 1990 to 2021 to assess its trends and disease burden.

The extracted data comprised the following dimensions: demographic characteristics such as age group (60+), sex, and year (1990–2021); geographical classification encompassing data from 204 countries/regions globally, including WHO member states, five SDI levels, and 21 GBD regions; and the socio-demographic index (SDI), which reflects the socioeconomic background and health conditions of regions, combining the total fertility rate under 25 years of age, mean years of schooling for individuals aged 15 years and older, and per capita lag-distributed income. Based on SDI, the 204 countries/regions were categorized into five development levels: low, low-middle, middle, high-middle, and high (7). The regional classification of the 21 GBD regions was determined based on epidemiological similarities and geographical proximity (8).

### 2.2 Analytical metrics

This study employed the following metrics to quantify the disease burden of IS in individuals aged 60 years and older: incidence rate, prevalence rate, mortality rate, and DALYs (disability-adjusted life years) rate estimates, along with their 95% uncertainty intervals (UI). Calculations were performed for ASIR, ASPR, ASMR, and ASDR, based on the age structure of the GBD standard world population (9).

### 2.3 Statistical methods

Data analysis was performed using Excel 2021 and R software version 4.3.3. Following data cleaning and organizing, statistical analyses and visualizations were performed using R packages, including dplyr, officer, and ggplot2. A *p*-value of <0.05 was considered statistically significant.

#### 2.3.1 Age group analysis

Based on the age grouping principles outlined in GBD 2021, subjects were categorized into the following eight age groups: 60–64, 65–69, 70–74, 75–79, 80–84, 85–89, 90–94, and 95+ years. The study analyzed the dynamics of IS burden variation across age groups and genders.

#### 2.3.2 Trend analysis

##### 2.3.2.1 Estimated annual percentage change

R software was employed to calculate and visualize the percentage changes in IS burden by age, sex, region, and country from 1990 to 2021. Trends in age-standardized rates were quantified using the Estimated Annual Percentage Change. An EAPC lower limit of the 95% confidence interval greater than 0 indicated an increasing trend, while less than 0 indicated a decreasing trend.

##### 2.3.2.2 Joinpoint regression model

The Joinpoint regression model was used to analyze temporal trends from 1990 to 2021, calculating the Average Annual Percent Change (AAPC) and Annual Percent Change (APC) for each time period. If the 95% confidence interval contained 0, the trend was considered stable. AAPC or APC significantly greater than or less than 0 indicated an upward or downward trend, respectively (10). Model fitting and AAPC/APC calculations were performed using Joinpoint software (version 4.9.0.0), with visualizations completed in R.

#### 2.3.3 Correlation analysis

Pearson correlation analysis was employed to assess the relationship between SDI and age-standardized IS rates among older adults: A positive r-value with *p* < 0.05 indicated a positive correlation; a negative r-value with *p* < 0.05 indicated a negative correlation; a *p*-value greater than 0.05 suggested no significant linear correlation, warranting further exploration of other relationships; r-value close to 1 indicated a strong positive correlation, close to −1 indicated a strong negative correlation, and close to 0 suggested a weak linear correlation, potentially influenced by other complex factors or requiring non-linear modeling.

#### 2.3.4 Decomposition and forecasting analysis

##### 2.3.4.1 Decomposition analysis

This method quantified the independent contributions of population growth, aging, and epidemiological changes to the IS burden among older adults, while controlling for other factors.

##### 2.3.4.2 Age-period-cohort analysis

The Age-Period-Cohort (APC) model assessed dynamic changes in IS risk influenced by age, period, and cohort factors. Based on a Poisson distribution, the model disaggregated variables into three dimensions to examine their effects on IS incidence and mortality in older adults (11).

##### 2.3.4.3 Bayesian age-period-cohort model

An extension of the age-period-cohort (APC) model that incorporates Bayesian Markov Chain Monte Carlo (MCMC) methods to address parameter estimation challenges arising from linear relationships among the three factors (12). This model was used to forecast the IS burden among older adults from 2022 to 2030.

## 3 Results

### 3.1 Global burden of IS in the elderly

In 2021, the global ASIR of IS among elderly individuals was 540.21 (95% UI: 405.64, 702.52) per 100,000 population, the ASPR was 4246.75 (95% UI: 3773.02, 4768.74) per 100,000 population, the ASMR was 333.80 (95% UI: 293.46, 364.19) per 100,000 population, and the ASDR was 5607.59 (95% UI: 5033.44, 6086.38) per 100,000 person-years. This indicates that in 2021, there were an estimated 5,706,996.97 (95% UI: 4,269,491.75, 7,444,063.44) new cases of IS among the elderly globally, with 2,875,384.11 (95% UI: 2,107,399.07, 3,805,862.27) cases in males and 2,831,612.86 (95% UI: 2,091,624.76, 3,731,970.50) cases in females.

The global number of existing IS cases among elderly individuals in 2021 was 45,524,895.79 (95% UI: 40,453,360.48, 51,105,265.87), including 23,212,809.42 (95% UI: 20,720,868.48, 25,985,058.36) in males and 22,312,086.37 (95% UI: 19,707,058.18, 25,226,719.64) in females. In the same year, 3,391,448.91 (95% UI: 2,992,926.36, 3,698,707.10) elderly individuals died from IS, including 1,651,235.44 (95% UI: 1,472,757.50, 1,830,612.26) in males and 1,740,213.47 (95% UI: 1,480,043.56, 1,938,004.81) in females.

The ASDR attributed to IS in 2021 amounted to 58,823,386.87 (95% UI: 52,918,664.55, 63,825,538.43) per 100,000 person-years, with 30,333,215.55 (95% UI: 27,340,806.16, 33,398,002.42) per 100,000 person-years in males and 28,490,171.31 (95% UI: 24,856,465.58, 31,614,973.57) per 100,000 person-years in females.

From 1990 to 2021, all estimated annual percentage changes (EAPCs) of disease burden indicators for IS among the elderly were negative, indicating an overall declining trend in the global burden of IS in this population (Table 1 for details).

**Table 1:** Age-standardized ischemic stroke burden results in the elderly aged ≥ 60 years for the global population, five SDI regions, and 21 GBD regions.

Location	Incidence			Prevalence			Deaths			DALYs		
	1990 (per 100,000 population, 95% UI)	2021 (per 100,000 population, 95% UI)	EAPCs (95% CI)	1990 (per 100,000 population, 95% UI)	2021 (per 100,000 population, 95% UI)	EAPCs (95% CI)	1990 (per 100,000 population, 95% UI)	2021 (per 100,000 population, 95% UI)	EAPCs (95% CI)	1990 (per 100,000 person-years,95% UI)	2021 (per 100,000 person-years,95% UI)	EAPCs (95% CI)
Global	656.87 (480.49, 863.89)	540.21 (405.64, 702.52)	−0.75 (−0.95, −0.55)	8771.64 (8030.60, 9425.26)	5607.59 (5033.44, 6086.38)	−1.66 (−1.88, −1.45)	4346.61 (3850.58, 4904.88)	4246.75 (3773.02, 4768.74)	−0.17 (−0.40, 0.06)	554.83 (499.31, 595.24)	333.80 (293.46, 364.19)	−1.82 (−1.99, −1.65)
SDI												
High SDI	629.01 (472.59, 813.54)	366.79 (282.25, 468.01)	−2.01 (−2.30, −1.72)	6407.99 (5814.66, 6812.45)	2584.44 (2235.39, 2860.33)	−3.27 (−3.54, −2.99)	4962.64 (4462.82, 5497.12)	4208.37 (3837.01, 4593.55)	−0.71 (−1.05, −0.37)	415.33 (367.26, 439.60)	148.48 (122.91, 162.71)	−3.65 (−3.85, −3.45)
High-middle SDI	866.86 (631.50, 1142.00)	710.81 (533.56, 928.51)	−0.74 (−0.96, −0.51)	13007.50 (12029.09, 13719.26)	7361.88 (6557.56, 8086.64)	−2.22 (−2.46, −1.97)	4512.54 (3955.99, 5132.76)	4772.83 (4206.62, 5405.06)	0.09 (−0.21, 0.38)	852.49 (777.82, 898.01)	454.99 (396.65, 502.35)	−2.35 (−2.56, −2.15)
Middle SDI	578.07 (411.05, 780.10)	624.88 (461.14, 822.23)	0.18 (−0.04, 0.39)	8175.70 (7294.81, 9213.00)	6518.62 (5761.99, 7211.17)	−0.79 (−1.05, −0.53)	3835.58 (3242.85, 4489.15)	4434.85 (3850.74, 5105.19)	0.44 (0.28, 0.60)	499.79 (441.07, 564.70)	391.42 (339.49, 435.93)	−0.82 (−1.07, −0.57)
Low-middle SDI	499.79 (361.40, 669.02)	450.92 (337.47, 583.13)	−0.44 (−0.53, −0.34)	7368.27 (6431.98, 8530.44)	6348.93 (5621.10, 7215.17)	−0.53 (−0.64, −0.42)	3226.27 (2736.94, 3756.49)	3109.16 (2698.91, 3545.24)	−0.15 (−0.33, 0.02)	444.15 (381.96, 515.33)	383.89 (336.13, 435.79)	−0.48 (−0.57, −0.39)
Low SDI	523.75 (379.44, 698.56)	454.03 (341.51, 588.38)	−0.52 (−0.57, −0.47)	7289.12 (6065.96, 9167.70)	6212.60 (5257.89, 7618.25)	−0.56 (−0.66, −0.47)	4075.81 (3580.62, 4610.00)	3603.29 (3223.01, 4005.57)	−0.47 (−0.53, −0.41)	431.43 (354.24, 545.34)	374.46 (312.46, 460.12)	−0.45 (−0.54, −0.37)
GBD 21 regions												
Andean Latin America	351.64 (264.01, 457.45)	248.97 (190.27, 317.73)	−1.20 (−1.30, −1.09)	3782.45 (3227.25, 4370.67)	2149.01 (1748.84, 2617.84)	−2.06 (−2.19, −1.94)	2892.52 (2657.90, 3127.49)	2456.91 (2288.73, 2629.47)	−0.61 (−0.66, −0.57)	238.34 (200.43, 278.07)	134.85 (108.02, 166.77)	−2.07 (−2.19, −1.95)
Australasia	566.59 (472.03, 664.46)	303.53 (238.44, 372.42)	−2.40 (−2.75, −2.06)	5228.20 (4634.15, 5710.90)	1726.53 (1441.26, 1956.45)	−3.83 (−4.00, −3.67)	4278.12 (4027.94, 4553.51)	3133.59 (2950.80, 3321.55)	−1.20 (−1.65, −0.74)	358.48 (309.34, 394.14)	110.77 (88.90, 125.29)	−4.04 (−4.13, −3.96)
Caribbean	428.14 (330.56, 541.48)	367.30 (286.46, 459.65)	−0.50 (−0.61, −0.39)	6044.46 (5471.26, 6562.33)	4328.90 (3745.52, 4952.42)	−1.05 (−1.11, −0.98)	3032.42 (2792.86, 3298.42)	2886.78 (2677.03, 3103.78)	−0.16 (−0.26, −0.06)	398.54 (358.88, 430.44)	272.52 (234.05, 311.61)	−1.18 (−1.25, −1.11)
Central Asia	769.80 (593.78, 971.71)	757.82 (597.87, 931.46)	0.03 (−0.09, 0.14)	10437.12 (9536.12, 11153.32)	9042.33 (8130.72, 9880.78)	−0.88 (−1.16, −0.60)	5019.84 (4581.99, 5474.94)	4854.00 (4495.57, 5244.09)	−0.09 (−0.11, −0.07)	604.14 (546.02, 647.19)	534.46 (474.43, 585.49)	−0.78 (−1.03, −0.53)
Central Europe	977.92 (762.63, 1216.99)	671.70 (534.34, 821.22)	−1.32 (−1.51, −1.14)	16421.39 (15462.90, 17111.64)	7802.84 (7060.10, 8417.89)	−2.75 (−3.00, −2.50)	5039.87 (4512.95, 5626.26)	4180.74 (3795.15, 4593.01)	−0.70 (−1.05, −0.34)	1085.08 (1009.58, 1130.92)	505.88 (449.99, 546.69)	−2.79 (−2.96, −2.61)
Central Latin America	457.80 (340.48, 599.50)	294.92 (223.37, 378.39)	−1.63 (−1.78, −1.48)	4345.28 (4058.29, 4566.98)	2274.93 (2019.51, 2533.20)	−2.27 (−2.41, −2.14)	3563.75 (3181.61, 3973.80)	2716.06 (2444.50, 3015.35)	−1.02 (−1.07, −0.96)	284.53 (262.35, 298.69)	142.26 (123.66, 159.32)	−2.36 (−2.48, −2.25)
Central Sub-Saharan Africa	703.33 (514.61, 933.68)	633.50 (477.81, 819.16)	−0.40 (−0.59, −0.22)	8452.01 (6462.86, 10929.58)	7646.80 (5637.22, 10315.06)	−0.47 (−0.56, −0.39)	5872.99 (5302.91, 6461.81)	5298.04 (4861.58, 5742.76)	−0.42 (−0.71, −0.13)	494.43 (368.45, 650.36)	459.59 (327.30, 636.57)	−0.39 (−0.47, −0.31)
East Asia	631.59 (435.55, 867.67)	853.99 (615.31, 1144.11)	0.91 (0.59, 1.22)	9200.67 (7864.80, 10754.36)	7983.02 (6704.97, 9196.73)	−0.47 (−0.87, −0.06)	4089.30 (3377.76, 4906.17)	5823.71 (4976.51, 6773.01)	1.18 (0.94, 1.43)	557.29 (471.74, 655.59)	480.62 (394.54, 561.69)	−0.48 (−0.93, −0.02)
Eastern Europe	1179.40 (829.90, 1608.98)	837.50 (612.98, 1112.81)	−1.20 (−1.35, −1.05)	19582.21 (18458.13, 20264.12)	10818.66 (9785.06, 11716.88)	−2.71 (−3.07, −2.34)	4642.68 (3928.86, 5396.23)	4294.56 (3673.09, 4958.56)	−0.29 (−0.53, −0.06)	1277.61 (1188.58, 1322.61)	687.92 (611.49, 748.56)	−2.82 (−3.12, −2.51)
Eastern Sub-Saharan Africa	623.98 (451.57, 833.11)	602.20 (449.93, 780.53)	−0.10 (−0.23, 0.04)	6802.12 (5532.12, 8474.22)	6114.90 (5071.81, 7275.19)	−0.44 (−0.48, −0.39)	5578.77 (4948.51, 6246.88)	5254.94 (4746.63, 5807.25)	−0.25 (−0.44, −0.06)	396.81 (315.97, 503.91)	356.24 (284.37, 433.10)	−0.43 (−0.48, −0.38)
High-income Asia Pacific	701.28 (489.51, 969.57)	334.23 (252.05, 430.80)	−2.99 (−3.49, −2.48)	7417.93 (6568.26, 8011.70)	2225.16 (1856.28, 2528.18)	−4.30 (−4.84, −3.76)	5646.51 (4963.13, 6367.62)	4094.08 (3663.02, 4567.21)	−1.26 (−1.83, −0.69)	486.97 (418.57, 526.45)	122.25 (95.92, 138.39)	−4.81 (−5.26, −4.36)
High-income North America	525.99 (364.02, 728.15)	305.06 (221.94, 411.20)	−1.84 (−2.01, −1.66)	3761.11 (3324.36, 4110.38)	2268.57 (1936.23, 2537.68)	−2.18 (−2.35, −2.00)	5009.69 (4305.48, 5752.27)	4652.02 (4121.50, 5203.79)	−0.55 (−0.76, −0.34)	229.06 (195.79, 246.61)	129.50 (105.11, 142.22)	−2.46 (−2.65, −2.27)
Oceania	475.33 (350.27, 630.78)	427.02 (326.59, 543.97)	−0.41 (−0.53, −0.30)	6266.23 (4890.17, 8094.92)	5293.53 (4221.09, 6840.61)	−0.65 (−0.91, −0.38)	4081.48 (3718.26, 4454.84)	3779.06 (3501.80, 4060.99)	−0.26 (−0.31, −0.21)	369.45 (282.42, 485.22)	310.00 (240.46, 410.16)	−0.68 (−0.98, −0.37)
South Asia	417.87 (292.15, 573.23)	343.62 (249.05, 457.58)	−0.87 (−0.98, −0.76)	5540.90 (4567.99, 6919.98)	4752.91 (4085.86, 5848.38)	−0.66 (−0.78, −0.53)	2414.86 (1932.38, 2945.48)	2220.47 (1829.82, 2646.66)	−0.28 (−0.64, 0.09)	328.30 (267.37, 410.46)	289.40 (246.03, 352.25)	−0.54 (−0.65, −0.43)
Southeast Asia	617.87 (450.51, 818.05)	614.11 (465.81, 786.31)	−0.06 (−0.19, 0.08)	9089.94 (7890.11, 10288.67)	8539.47 (7241.52, 9763.51)	−0.16 (−0.37, 0.06)	4607.40 (3995.77, 5264.32)	4685.19 (4154.51, 5248.90)	0.04 (−0.09, 0.17)	548.43 (466.38, 628.93)	515.51 (433.22, 591.58)	−0.11 (−0.28, 0.06)
Southern Latin America	552.18 (426.56, 699.85)	333.94 (259.75, 413.50)	−1.84 (−2.09, −1.58)	6947.40 (6266.91, 7514.10)	2736.93 (2428.29, 3011.04)	−2.75 (−3.06, −2.44)	4581.24 (4247.24, 4935.24)	3342.88 (3136.25, 3559.38)	−1.13 (−1.46, −0.79)	445.50 (395.95, 482.85)	164.68 (142.60, 181.42)	−2.83 (−3.06, −2.60)
Southern Sub-Saharan Africa	713.21 (497.58, 974.72)	766.41 (553.91, 1024.91)	0.13 (−0.12, 0.39)	6217.13 (5162.19, 7109.52)	7639.48 (6840.18, 8435.19)	0.74 (0.47, 1.01)	6796.33 (5753.20, 7958.97)	6277.04 (5405.76, 7242.31)	−0.32 (−0.62, −0.02)	361.12 (290.49, 418.10)	465.92 (410.72, 517.66)	0.91 (0.62, 1.20)
Tropical Latin America	670.52 (457.27, 937.54)	394.88 (280.87, 530.73)	−1.81 (−2.18, −1.44)	9377.83 (8599.59, 9877.56)	3606.57 (3211.04, 3877.98)	−2.94 (−3.30, −2.58)	4380.96 (3674.76, 5188.80)	3227.76 (2743.96, 3766.42)	−1.11 (−1.44, −0.77)	606.23 (542.97, 642.62)	225.42 (193.65, 244.21)	−2.95 (−3.24, −2.66)
Western Europe	662.14 (520.37, 817.91)	339.93 (278.17, 407.70)	−2.39 (−2.66, −2.12)	7034.47 (6360.43, 7457.28)	2086.81 (1781.82, 2314.46)	−4.14 (−4.39, −3.89)	4468.33 (4083.31, 4876.29)	3458.62 (3241.40, 3685.68)	−0.90 (−1.28, −0.52)	490.27 (431.70, 520.32)	130.72 (106.94, 143.73)	−4.49 (−4.65, −4.32)
Western Sub-Saharan Africa	554.26 (395.35, 747.26)	508.61 (378.88, 660.33)	−0.24 (−0.29, −0.19)	9588.98 (7621.69, 12300.50)	8515.89 (7136.36, 10102.14)	−0.37 (−0.74, 0.01)	5326.32 (4667.72, 6052.78)	5046.92 (4522.36, 5619.60)	−0.22 (−0.32, −0.12)	577.65 (449.60, 747.03)	517.18 (429.04, 617.43)	−0.35 (−0.70, 0.00)

### 3.2 Regional burden of IS in the elderly (SDI)

In the five SDI regions, the ASIR: 710.81 (95% UI: 533.56,928.51) per 100,000 population, ASPR: 4772.83 (95% UI: 4206.62,5405.06) per 100,000 population, ASMR: 454.99 (95% UI: 396.65,502.35) per 100,000 population, and ASDR: 7361.88 (95% UI: 6557.56,8086.64) per 100,000 person-years for IS in the elderly in the High-middle SDI region in 2021 were the highest. In contrast, the High SDI region had the lowest ASIR: 366.79 (95% UI: 282.25,468.01) per 100,000 population, ASMR: 148.48 (95% UI: 122.91,162.71) per 100,000 population, and ASDR: 2584.44 (95% UI: 2235.39,2860.33) per 100,000 person-years, while the Low-middle SDI region had the lowest ASPR: 3109.16 (95% UI: 2698.91,3545.24) per 100,000 population (Table 1).

**Figure 1:**
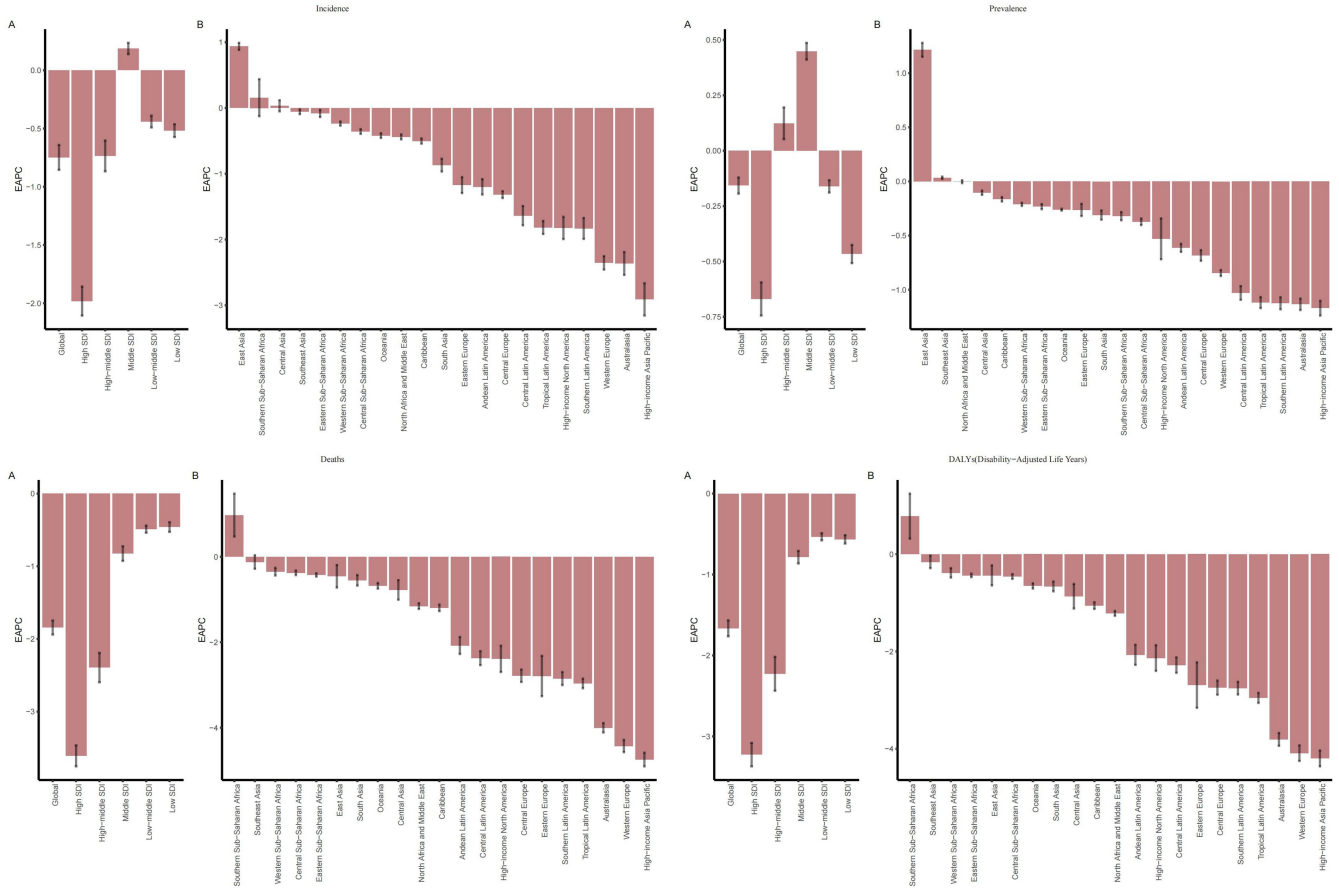
EAPC of IS among elderly patients globally, across 5 SDI regions, and 21 GBD regions (1990–2021): **(A)** EAPCs for 5 SDIs; **(B)** EAPCs for 21 regions.

In terms of temporal trends, except for the ASIR (EAPC = 0.18, 95%CI: −0.04, 0.39) and ASPR (EAPC = 0.44, 95%CI: 0.28, 0.60) in the Middle SDI region, and the ASPR (EAPC = 0.09, 95%CI: −0.21, 0.38) in the High-middle SDI region showing an increasing trend, the disease burden of IS in the elderly decreased in all other SDI regions. These findings may indicate that amid socioeconomic transitions—characterized by rapid urbanization, an accelerated pace of work and life, and dramatic changes in dietary and physical activity patterns—the exposure to cardiovascular risk factors among the elderly has been exacerbated to some extent. In high-SDI regions, elderly patients with IS have benefited from systematic early screening programs, standardized rapid diagnostic protocols, and the ongoing implementation and optimization of integrated public health interventions. Consequently, between 1990 and 2021, the ASIR (EAPC = −2.01, 95%CI: −2.30, −1.72), ASPR (EAPC = −0.71, 95%CI: −1.05, −0.37), ASMR (EAPC = −3.65, 95%CI: −3.85, −3.45), and ASDR (EAPC = −3.27, 95%CI: −3.54, −2.99) exhibited the most pronounced declining trends, all of which were significantly more favorable than those observed in regions with other SDI levels (Figure 1; Table 1).

**Figure 2:**
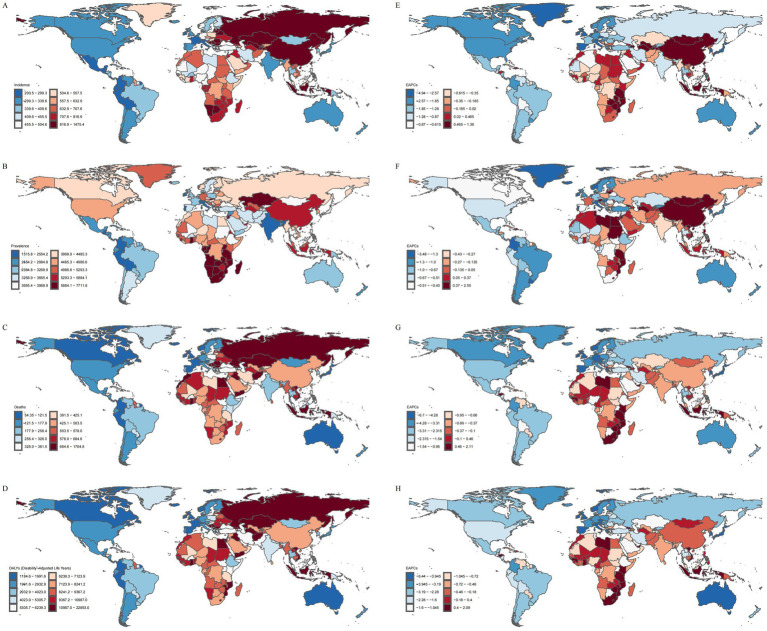
Disease burden of IS in elderly patients across 204 countries and regions worldwide in 2021. **(A)** ASIR. **(B)** ASPR. **(C)** ASMR. **(D)** ASDR. **(E)** EAPC of ASIR. **(F)** EAPC of ASPR. **(G)** EAPC of ASMR. **(H)** EAPC of ASDR.

Among the 21 GBD regions, East Asia had the highest ASIR: 853.99 (95% UI: 615.31, 1144.11) per 100,000 population in 2021, Southern Sub-Saharan Africa had the highest ASPR: 6277.04 (95% UI: 5405.76, 7242.31), and Eastern Europe had the highest ASMR: 687.92 (95% UI: 611.49, 748.56) per 100,000 population and ASDR: 10818.66 (95% UI: 9785.06, 11716.88) per 100,000 person-years. In contrast, Andean Latin America had the lowest ASIR: 248.97 (95% UI: 190.27, 317.73) per 100,000 population, South Asia had the lowest ASPR: 2220.47 (95% UI: 1829.82, 2646.66), and Australasia had the lowest ASMR: 110.77 (95% UI: 88.90, 125.29) per 100,000 population and ASDR: 1726.53 (95% UI: 1441.26, 1956.45) per 100,000 person-years (Figures 2A–D; Table 1).

The region with the greatest gender disparity was High-income Asia Pacific showed the greatest gender disparity in ASIR, ASPR, and ASDR, with male rates approximately 0.76 times, 0.97 times, and 0.99 times higher than female rates, respectively. In terms of mortality, East Asia exhibited the greatest gender disparity, with male mortality about 0.78 times higher than female mortality.

The highest male values were found in East Asia, where the male ASIR and ASPR were the highest, with the male ASIR being 1003.93 (95% UI: 715.10, 1356.87) per 100,000 population, and the male ASPR being 6320.69 (95% UI: 5452.88, 7280.59) per 100,000 population. In Eastern Europe, the highest male ASMR and ASDR were recorded, with the male ASMR being 754.20 (95% UI: 667.52, 831.43) per 100,000 population and the male ASDR being 12849.15 (95% UI: 11498.58, 14147.18) per 100,000 person-years.

The highest female values were observed in Southern Sub-Saharan Africa, where the female ASIR and ASPR were the highest. The female ASIR was 822.02 (95% UI: 585.87, 1112.83) per 100,000 population, and the female ASPR was 6928.38 (95% UI: 5952.55, 7952.15) per 100,000 population, Southern Sub-Saharan Africa’s elderly women face significant disadvantages in accessing quality healthcare services. Conversely, elderly men are markedly more likely to receive a diagnosis and obtain appropriate treatment, underscoring the presence of pronounced gender disparities (13). In Eastern Europe, the highest female ASMR and ASDR were found, with the female ASMR being 638.57 (95% UI: 555.70, 708.18) per 100,000 population, and the female ASDR being 9492.92 (95% UI: 8381.71, 10463.60) per 100,000 person-years. Eastern Europe, as a typical high-middle SDI region, exhibits the highest ASMR and ASDR for IS among the elderly, in both males and females. The region is currently undergoing a profound socioeconomic transition, a phase that has directly impacted the allocation and accessibility of primary public health resources. Moreover, entrenched cultural traditions characterized by high alcohol consumption and other unhealthy lifestyle practices, coupled with a relatively weak emphasis on preventive healthcare, have further exacerbated the overall disease burden (14) (Supplementary Figure 1).

With respect to temporal trends, except for a few regions, the disease burden in different GBD regions has been alleviating. Among these, East Asia showed the largest increases in ASIR (EAPC = 0.91, 95%CI: 0.59, 1.22) and ASPR (EAPC = 1.18, 95%CI: 0.94, 1.43), and Southern Sub-Saharan Africa showed the largest increases in ASMR (EAPC = 0.91, 95%CI: 0.62, 1.20) and ASDR (EAPC = 0.74, 95%CI: 0.47, 1.01). These regions are facing a growing burden of IS in the elderly, posing a tremendous challenge to local healthcare systems. Meanwhile, the disease burden in the High-income Asia Pacific region has decreased significantly, with a decline in ASIR (EAPC = −2.99, 95%CI: −3.49, −2.48), ASPR (EAPC = −1.26, 95%CI: −1.83, −0.69), ASMR (EAPC = −4.81, 95% CI: −5.26, −4.36), and ASDR (EAPC = −4.30, 95%CI: −4.84, −3.76). In the High-income Asia Pacific region, a relatively high level of education has endowed the public with a strong awareness of IS risks and the importance of early intervention. This awareness is largely attributable to extensive and sustained health education and promotional initiatives, which facilitate the timely management of associated risk factors. In contrast, several developing countries in East Asia exhibit comparatively insufficient performance in this regard (15) (Figures 1, 2E–H; Table 1).

### 3.3 National and regional burden of IS in the elderly

**Figure 3:**
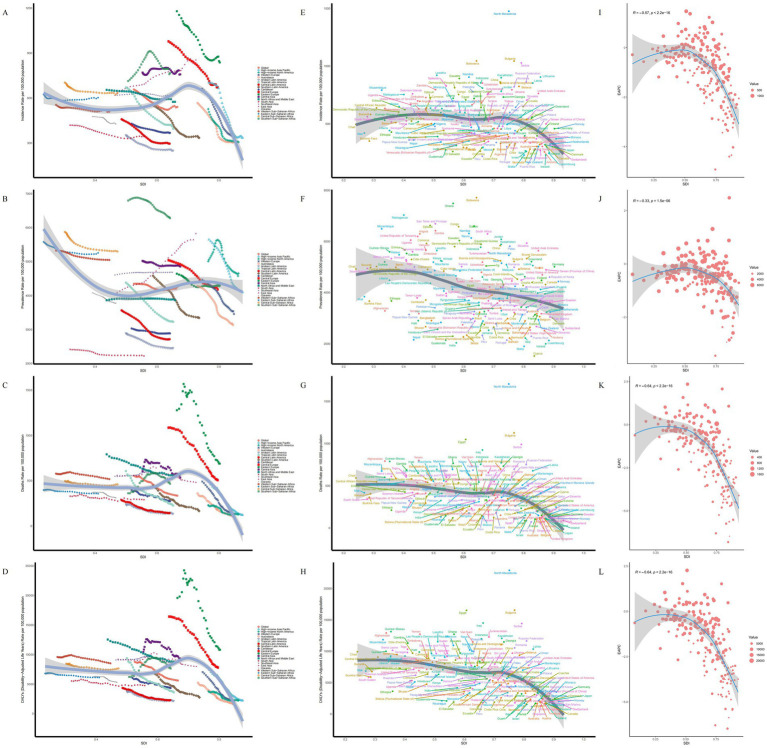
SDI analysis results. **(A)** ASIR in 21 regions. **(B)** ASPR in 21 regions. **(C)** ASMR in 21 regions. **(D)** ASDR in 21 regions. **(E)** ASIR in 204 countries. **(F)** ASPR in 204 countries. **(G)** ASMR in 204 countries. **(H)** ASDR in 204 countries. **(I)** EAPC of ASIR. **(J)** EAPC of ASPR. **(K)** EAPC of ASMR. **(L)** EAPC of ASDR.

In 2021, North Macedonia reported the highest ASIR, ASMR, and ASDR for IS in the elderly, with values of 1475.40 (95% UI: 1181.13, 1791.12) per 100,000 population, 1704.84 (95% UI: 1429.76, 1997.88) per 100,000 population, and 22893.04 (95% UI: 19025.21, 26976.17) per 100,000 person-years, respectively. Botswana reported the highest ASPR, which was 7711.78 (95% UI: 7086.80, 8373.89) per 100,000 population. In contrast, Puerto Rico exhibited the lowest ASIR and ASDR, with values of 200.52 (95% UI: 150.86, 261.45) per 100,000 population and 1184.56 (95% UI: 974.15, 1385.75) per 100,000 person-years, respectively. The Republic of Cyprus reported the lowest ASPR, recorded at 1515.82 (95% UI: 1322.86, 1754.27) per 100,000 population. The Republic of Singapore reported the lowest ASMR, at 54.35 (95% UI: 43.43, 63.15) per 100,000 population (Figures 3E–H).

Longitudinal trend analysis revealed that the disease burden of IS decreased in 174 countries, whereas 30 countries exhibited an increasing trend in the disease burden among the elderly. These countries included: Burkina Faso, Cabo Verde, Cameroon, Chad, Democratic Republic of Timor-Leste, Dominican Republic, Eswatini, Gambia, Georgia, Ghana, Guinea, Guinea-Bissau, Kenya, Lesotho, Libya, Malawi, Montenegro, Mozambique, Niger, Republic of Honduras, Republic of Indonesia, Republic of Kiribati, Sao Tome and Principe, Socialist Republic of Vietnam, South Africa, Turkmenistan, United Arab Emirates, United Republic of Tanzania, Zambia, and Zimbabwe.

Among these, the Republic of Tajikistan experienced the largest increase in ASIR (EAPC = 1.36, 95%CI: 1.24, 1.48), the Republic of Lithuania reported the largest increase in ASPR (EAPC = 2.55, 95%CI: 1.88, 3.23), Lesotho recorded the largest increase in ASMR (EAPC = 2.11, 95%CI: 1.82, 2.40), and ASDR (EAPC = 2.09, 95%CI: 1.83, 2.35). The Portuguese Republic had the largest decrease in ASIR (EAPC = −4.94, 95%CI: −5.26, −4.62) and ASPR (EAPC = −3.48, 95%CI: −3.86, −3.10), while the Republic of Estonia exhibited the largest decrease in ASMR (EAPC = −6.70, 95%CI: −7.17, −6.23) and ASDR (EAPC = −6.44, 95%CI: −6.94, −5.93). Over the past 30 years, the heterogeneity of the global IS burden among the elderly has been shaped not only by biological mechanisms but also by a combination of factors, including socioeconomic status (16), exposure to industrial pollution (17), and the efficiency of stroke care systems (18). These multifaceted influences have led to the emergence of distinct IS burden profiles across different countries.

### 3.4 Age-gender-time association analysis of IS in the elderly

**Figure 4:**
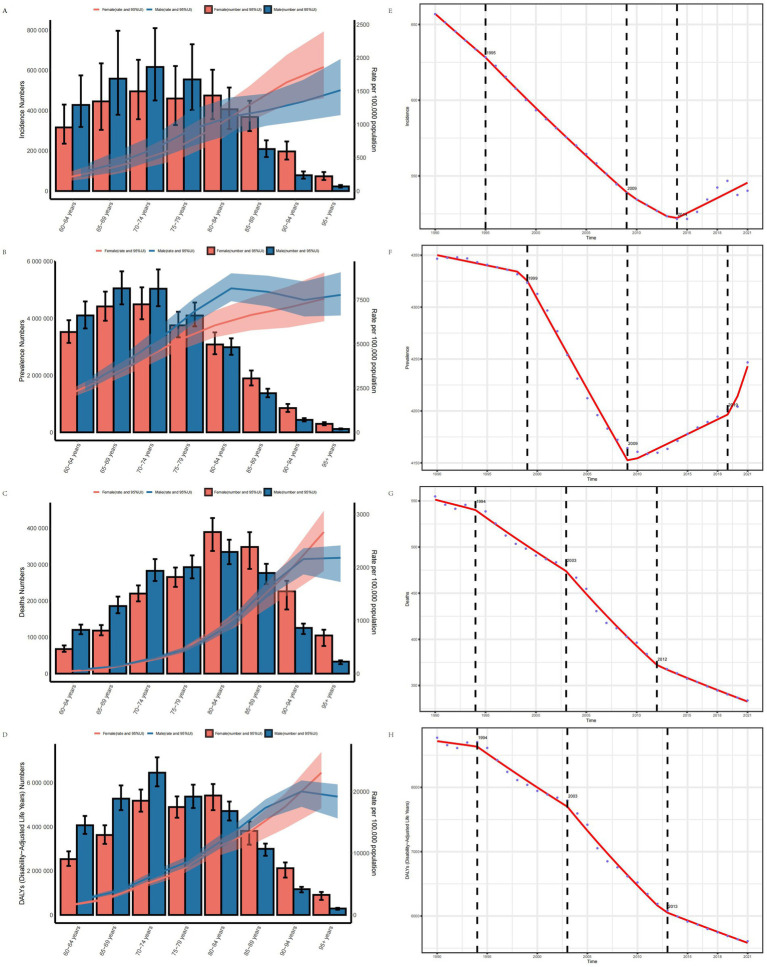
Age-gender trend and Joinpoint regression analysis of disease burden in elderly patients with IS. **(A)** ASIR. **(B)** ASPR. **(C)** ASMR. **(D)** ASDR. **(E)** Joinpoint analysis of ASIR. **(F)** Joinpoint analysis of ASPR. **(G)** Joinpoint analysis of ASMR. **(H)** Joinpoint analysis of ASDR.

The age-gender association analysis shows that with increasing age, the ASIR, ASPR, ASMR and ASDR of IS in males and females aged 60 and above in 2021 all exhibited an upward trend. In terms of ASIR, ASPR, ASMR, and ASDR, males generally exhibited higher values than females. However, in the 80–84 age group, the disease burden in females began to surpass that in males. Overall, the disease burden in males was higher than that in females across most indicators (Figures 4A–D).

The age-time association analysis shows that globally, the ASIR, ASPR, ASMR, and ASDR of IS in individuals aged 60 and above remained relatively stable from 1990 to 2021, followed by a significant downward trend over time. However, the ASPR among individuals aged 80 and above has exhibited an upward trend in recent years, reflecting the evolving age-time association (Supplementary Figure 2).

The gender-time association analysis indicates that from 1990 to 2021, the ASIR, ASPR, ASMR, and ASDR of IS in males aged 60 and above were generally higher than in females on a global scale. All indicators showed an increase over time, with a more substantial rise observed in males (Supplementary Figure 2).

### 3.5 Trends in the disease burden of IS in the elderly

Joinpoint regression analysis indicates that from 1990 to 2021, the ASIR, ASPR, ASMR, and ASDR of IS in the elderly exhibited an overall downward trend globally. Specifically, the annual average percent change (AAPC) for ASIR was −3.598 (95% CI: −3.794, −3.403), for ASPR was −3.438 (95% CI: −3.825, −3.052), for ASMR was −7.072 (95% CI: −7.377, −6.768), and for ASDR was −101.282 (95% CI: −105.970, −96.595). Significant changes in the ASIR occurred in 1995, 2009, and 2014, while change in ASPR occurred in 1999, 2009, and 2019. Significant shifts in the ASMR and ASDR were observed in 1994, 2003, and 2012, with ASDR notably changing in 2013 (Figures 4E–H).

### 3.6 Association between disease burden of IS in the elderly and SDI

In 2021, across 21 GBD regions globally, the SDI exhibited a negative linear relationship with the ASIR (*ρ* = −0.0847, *p* = 2.471e-02), ASPR (*ρ* = −0.0784, *p* = 3.766e-02), ASMR (*ρ* = −0.2540, *p* = 9.484e-12), and ASDR (ρ = −0.2855, *p* = 1.471e-14) of IS in the elderly. Specifically, as SDI increased, the disease burden of IS in the elderly exhibited a gradual downward trend (Figures 3I–L). However, East Asia represented a notable exception, where both the ASIR and ASPR increased as SDI rose. The slopes of the curves in certain regions were steeper than in others, suggesting that SDI-related factors (e.g., education level, wealth, and fertility rate) have a more pronounced impact on the disease burden in these areas (Figures 3A–D).

Among 204 countries, a significant negative linear relationship was also observed between SDI and the ASIR (ρ = −0.3401, *p* = 7.601e-07), ASPR (ρ = −0.3185, *p* = 3.869e-06), ASMR (ρ = −0.5265, *p* = 0.000e+00), and ASDR (ρ = −0.5512, p = 0.000e+00) of IS. As the SDI increases, the disease burden of IS among the elderly in various countries shows a gradual decline. However, despite North Macedonia and Bulgaria having comparable SDI levels, North Macedonia exhibits a heavier disease burden. This divergence is attributable to asymmetries in the enforcement of health policies, the level of investment in disease prevention, and the efficiency and integration of healthcare systems, which result in markedly different IS burdens among the elderly between the two countries (19). Additionally, the EAPC of the ASIR, ASPR, ASMR, and ASDR also decreased with increasing SDI, indicating that countries with higher SDI tend to exhibit a lower disease burden of IS in the elderly (Figures 3E–H).

### 3.7 Age-period-cohort analysis of disease burden in elderly patients with IS

The results of the age-period-cohort analysis of ASIR, ASPR, ASMR, and ASDR in elderly patients with IS reveal consistent trends.

**Figure 5:**
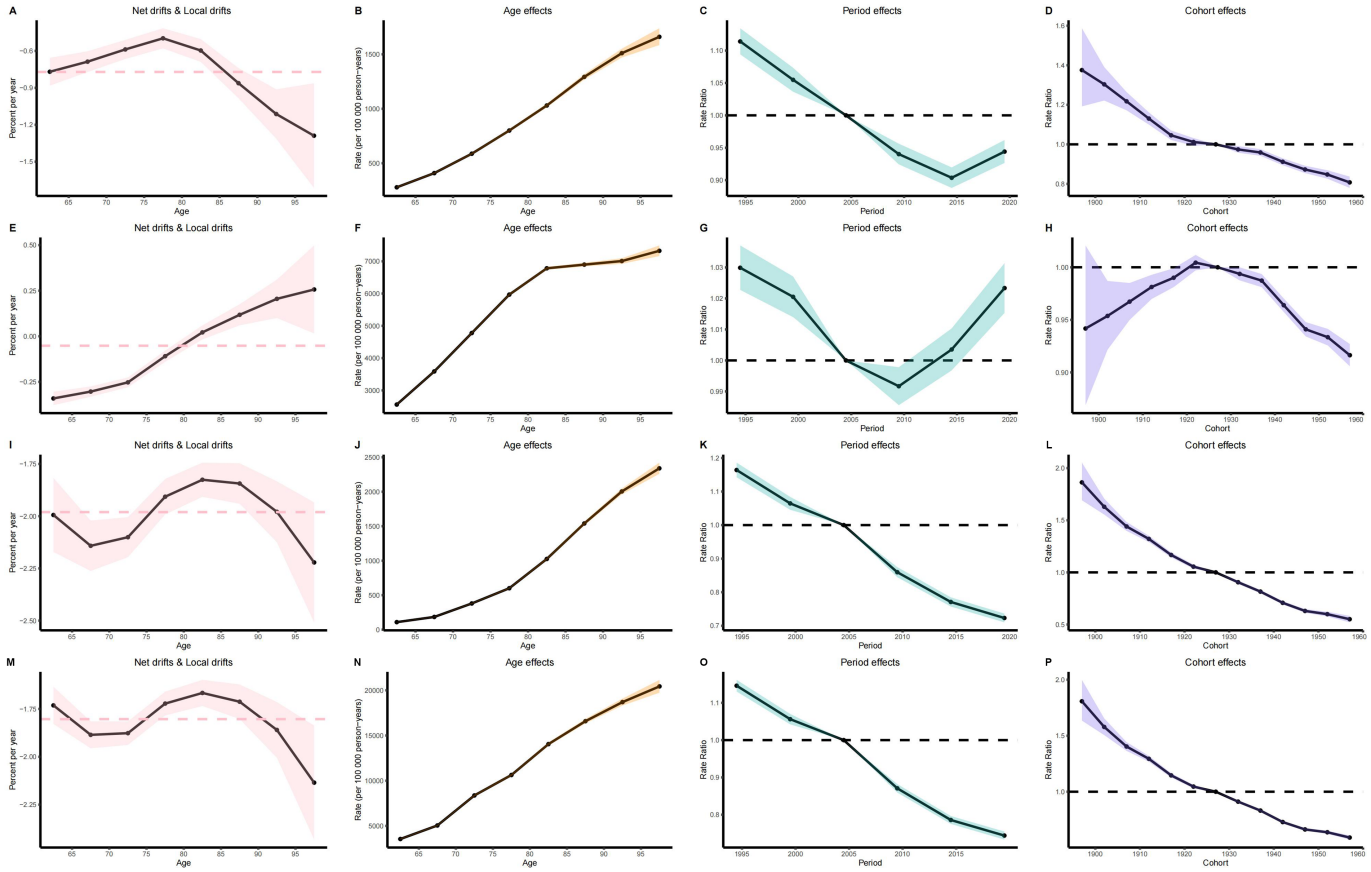
Age-period-cohort analysis results. **(A)** Net drift and local drift of ASIR. **(E)** Net drift and local drift of ASPR. **(I)** Net drift and local drift of ASMR. **(M)** Period effect of ASDR. **(B)** Age effect of ASIR. **(F)** Age effect of ASPR. **(J)** Age effect of ASMR. **(N)** Age effect of ASDR. **(C)** Period effect of ASIR. **(G)** Period effect of ASPR. **(K)** Period effect of ASMR. **(O)** Period effect of ASDR. **(D)** Cohort effect of ASIR. **(H)** Cohort effect of ASPR. **(L)** Cohort effect of ASMR. **(P)** Cohort effect of ASDR.

The age effect analysis indicates that as age increases, the disease burden of IS in elderly patients demonstrates a significant upward trend (Figures 5B,F,J,N). Correspondingly, the annual change in IS burden was modest at ages 60–70, accelerated or reached an inflection point between 70 and 85, and attained its greatest amplitude above 85, exhibiting an overall nonlinear drift of initial stability followed by marked intensification, underscoring the need to prioritize the oldest-old population (Figures 5A,E,I,M).

The period effect analysis shows that from 1990 to 2021, the global disease burden of elderly patients with IS gradually declined over time. However, the ASIR of IS in 2014 and the ASPR in 2009 began to exhibit an upward trend (Figures 5C,G,K,O).

The cohort effect analysis for the period from 1900 to 1960 demonstrates that the disease burden in elderly patients with IS significantly decreased in later-born cohorts relative to earlier-born cohorts. Elderly individuals born before 1922 generally exhibited higher ASPR, and this trend began to decline after 1922 (Figures 5D,H,L,P).

### 3.8 Decomposition analysis of ischemic stroke burden in elderly patients

**Figure 6:**
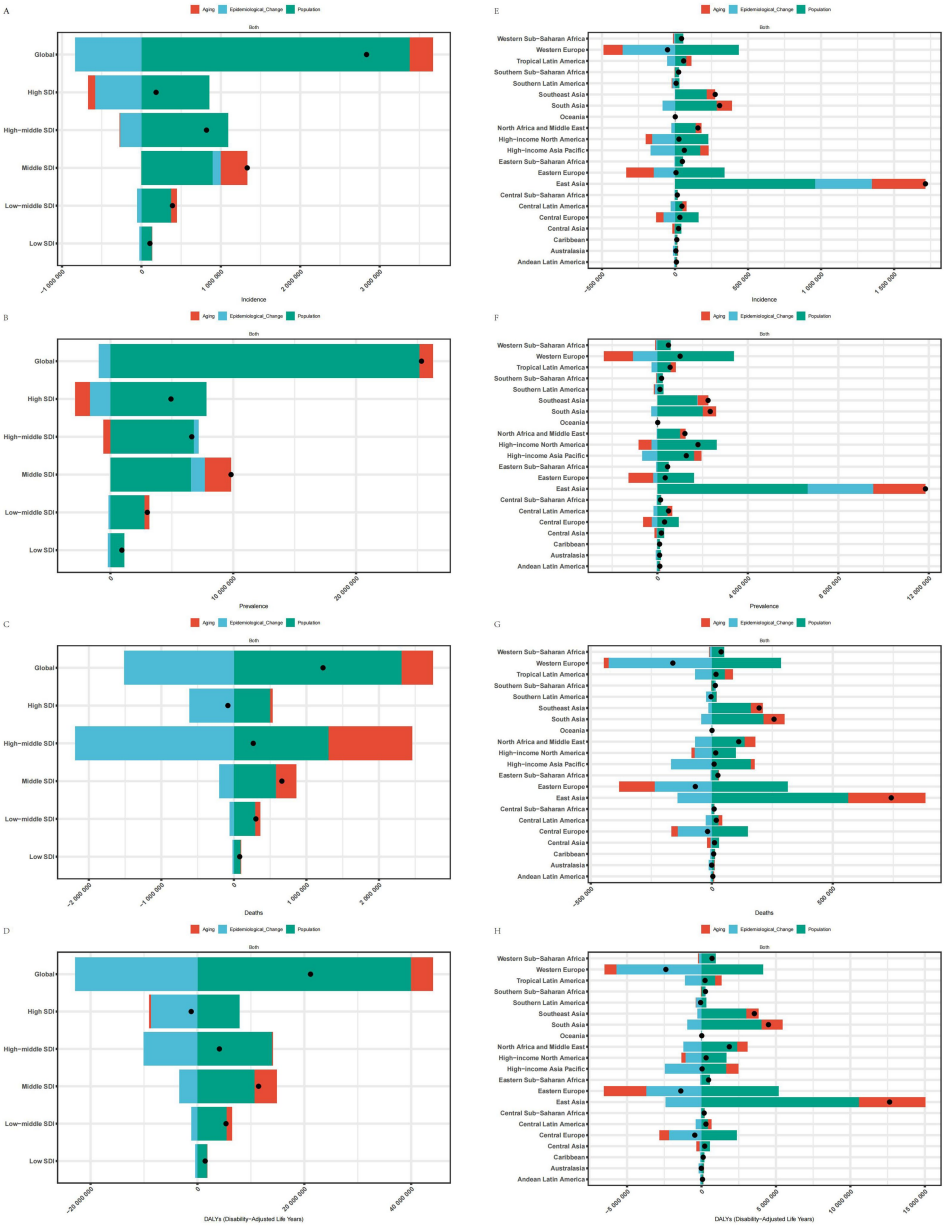
Decomposition analysis of SDI and 21 regions. **(A)** Decomposition analysis of ASIR for SDI. **(B)** Decomposition analysis of ASPR for SDI. **(C)** Decomposition analysis of ASMR for SDI. **(D)** Decomposition analysis of ASDR for SDI. **(E)** Decomposition analysis of ASIR for 21 regions. **(F)** Decomposition analysis of ASPR for 21 regions. **(G)** Decomposition analysis of ASMR for 21 regions; **(H)** Decomposition analysis of ASDR for 21 regions.

The decomposition analysis investigates the contribution of three factors—aging, epidemiological changes, and population growth—to the disease burden. Specifically, in the five SDI regions and 21 GBD regions where the disease burden of IS in older adults decreased, favorable epidemiological changes significantly reduced the ASMR, ASDR, and ASIR in High SDI, including Western Europe, Eastern Europe, Central Europe, Australasia, and Southern Latin America. Additionally, aging contributed to the reduction of the AMDR in the Australasia region and decreased the ASMR and ASDR in Western Europe, Eastern Europe, and Central Europe. Furthermore, population growth is the primary driver behind the increasing disease burden of IS among the elderly. The expansion of the population base, combined with an aging demographic, has resulted in a rising absolute number of cases and associated health losses, even as age-standardized indicators show a decline. At the global level and in other regions, population growth, aging, and certain adverse epidemiological changes collectively exacerbated the disease burden of IS in older adults, with the Middle SDI and East Asia regions experiencing the most pronounced burden (Figure 6).

### 3.9 Predictive analysis of disease burden for elderly patients with IS (BAPC)

The predictive analysis suggests that between 2022 to 2030, the global disease burden of IS among elderly individuals will continue to decline. By 2030, the ASIR, ASPR, ASMR, and ASDR for elderly IS patients will decrease to 505.80 (95% CI: 472.08, 539.51) per 100,000, 4100.58 (95% CI: 3927.72, 4273.44) per 100,000, 290.57 (95% CI: 265.41, 315.73) per 100,000, and 4977.57 (95% CI: 4557.08, 5398.06) per 100,000 person-years, respectively (Supplementary Figure 1). Furthermore, by 2030, the number of new IS patients aged 60 and older will increase by 7,236,190.40 (95% CI: 6,753,811.11, 7,718,569.69), bringing the total number of elderly IS patients to 58,664,794.72 (95% CI: 56,191,740.32, 61,137,849.11). Additionally, 4,157,052.07 (95% CI: 3,797,030.52, 4,517,073.62) per 100,000 individuals aged 60 and above are projected to die from IS, resulting in a loss of 71,211,504.28 (95% CI: 65,195,777.01, 77,227,231.56) life years (Supplementary Figure 3). Moreover, our predictive analysis across different age groups reveals a significant downward trend in the disease burden of IS, with the trend being more pronounced in older age groups.

### 3.10 Health inequality analysis

**Figure 7:**
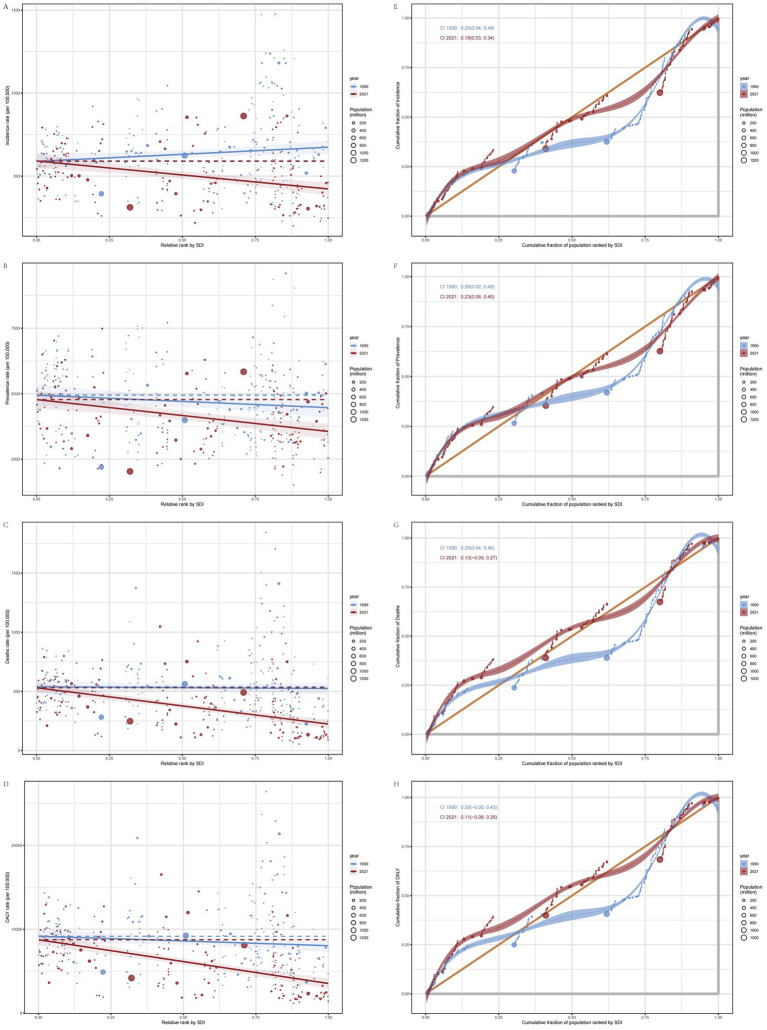
SII (the slope index of inequality) and CII (the concentration index of inequality) plots. **(A)** SII plot for ASIR. **(B)** SII for ASPR. **(C)** SII plot for ASMR. **(D)** SII plot for ASDR. **(E)** CII plot for ASIR. **(F)** CII plot for ASPR. **(G)** CII plot for ASMR. **(H)** CII plot for ASDR.

The slope index of inequality (SII) is an absolute metric used to quantify health disparities by comparing the differences between the poorest and wealthiest segments, while accounting for the proportional distribution across all socioeconomic strata. A higher SII indicates more pronounced health inequalities among different socioeconomic groups, whereas values approaching zero signify a more equitable distribution of health status (20). Over time, the ASIR for low SDI countries has gradually increased, while the ASIR for high SDI countries has decreased relatively, changing from 85.47 (95% CI: 5.34, 165.60) in 1990 to −168.29 (95% CI: −240.75, −95.83) in 2021. Simultaneously, the primary disease burden of IS among elderly individuals globally was concentrated in lower SDI regions both in both 1990 and 2021. The ASPR, ASMR, and ASDR worsened from −479.27 (95% CI: −1066.77, 108.22) to −1217.23 (95% CI: −1754.28, −680.18), from −14.52 (95% CI: −109.63, 80.60) to −306.37 (95% CI: −377.09, −235.66), and from −1129.79 (95% CI: −2622.32, 362.74) to −5209.82 (95% CI: −6313.15, −4106.48), respectively. This indicates an increase in the inequality in the disease burden, measured by the Slope Index of Inequality (SII), for IS among elderly individuals in lower SDI countries. There is a heavier burden in low SDI countries and a growing disparity between low and high SDI countries (Figures 7A–D).

The concentration index of inequality (CII) is a relative measure that utilizes a concentration curve to quantitatively characterize the distribution of IS across the entire socioeconomic spectrum. Its methodology is analogous to that used for evaluating income distribution concentration; however, it emphasizes health indicators rather than economic income. A positive CII value indicates that health outcomes (disease burden) are predominantly concentrated among individuals with higher SDI, whereas a negative value suggests that these outcomes are more concentrated among those with lower SDI. The closer the CII value is to zero, the more equitable the distribution (21). The CII for the ASIR, ASPR, ASMR, and ASDR of elderly IS patients globally indicate that, compared to 1990, the CII values for 2021 are smaller. This reduction in CII values demonstrates a decrease in the inequality of disease burden among elderly populations across different economic levels globally, resulting in a more equitable distribution. Furthermore, the CII values are positive, indicating that the disease burden for elderly IS patients is more concentrated in populations with higher SDI. Additionally, the burden is shifting toward regions with higher SDI (Figures 7E–H).

Research demonstrates that in contexts of pronounced wealth disparity, policymakers should employ both the SII and CII to gain a comprehensive understanding of health inequality. Given that these indices emphasize different dimensions, when health disparities are particularly extreme in certain populations (e.g., in situations with exceptionally wide wealth gaps), the SII and CII may yield divergent results: the SII is more effective at capturing the absolute differences between extreme groups, while the CII highlights the relative imbalance across the overall distribution (22).

## 4 Discussion

The findings from the GBD 2021 study reveal substantial variations in ASIR and ASPR of IS among the elderly across 21 GBD regions and 204 countries. However, the disparities in ASMR and ASDR are comparatively smaller. A study on the global burden of cardiovascular diseases suggests that the trend in DALYs correlates closely with mortality rates, reflecting the substantial contribution of premature death to these metrics. This pattern is consistent across various countries and populations, especially in the context of limited non-fatal outcomes (23). We have presented the most recent statistical data on the health indicators for IS in the elderly, including ASIR, ASPR, ASMR and ASDR, and elucidated the trends from 1990 to 2021. We found that, despite the increasing global elderly population, the disease burden of IS has generally decreased from 1990 to 2021, aligning with the findings of Zhang et al. (24). The reduction in the disease burden of IS among elderly patients mirrors significant advances in medical technology (such as thrombolysis and thrombectomy), the widespread use of preventive drugs like antithrombotic medications, and public health initiatives targeting modifiable risk factors such as hypertension and smoking. These measures, in conjunction with enhanced critical care systems, have not only reduced elderly mortality but also enhanced the prognosis of elderly stroke patients (25). Although advancements in medical technologies and comprehensive stroke management have significantly improved both treatment outcomes and preventive strategies, their overall effectiveness remains contingent upon timely access to care. Relative evidence indicates that many elderly individuals lack sufficient awareness of key stroke symptoms such as facial drooping, unilateral limb weakness, and speech disturbances, which is particularly pronounced among the older elderly population (26). These symptoms are frequently misattributed to the natural aging process or overlooked due to feelings of shame; inadequate recognition and delayed response among the elderly, their caregivers, and family members often lead to pre-hospital delays (27, 28). Consequently, the likelihood of receiving time-sensitive therapies such as thrombolysis or thrombectomy is reduced, thereby contributing to an underestimation of the true incidence of stroke (29). Targeted measures that encompass public education and multimedia campaigns—including FAST (Face, Arms, Speech, Time) and Stroke 1-2-0 (1: Face asymmetry, 2: Arm weakness, 0: Speech difficulty) campaign, has been associated with improved stroke recognition and timely medical intervention. Notably, these educational interventions exert a direct influence on elderly individuals while also indirectly enhancing stroke symptom recognition through the dissemination of information by family members and broader community networks, as evidenced by instances where students transmit critical stroke-related knowledge to their relatives (30, 31). Cumulatively, geographically tailored stroke education systems constitute a viable strategy for mitigating disability-adjusted life years and case fatality rates in elderly ischemic stroke populations worldwide.

In terms of disease burden indicators, males generally demonstrate elevated rates compared to females, as they have a relatively higher proportion of smoking and alcohol consumption, which may increase the risk of IS. However, in the 80–84 age group, the disease burden of IS in females starts to surpass that of males. Males face more significant cardiovascular challenges, with a higher incidence of diseases like hypertension, which are often more severe and increase the risk of premature death. This results in a higher number of male deaths from IS before the age of 80, accompanied by a relatively lower disease burden (5).

As age increases, the incidence, prevalence, mortality, and DALYs of IS in elderly individuals demonstrate an increasing trend. Compared with younger individuals, aging leads to thickening of vascular walls, reduced elasticity, and alterations in hemodynamics, all of which increase the risk of IS. Furthermore, the prognosis is poorer for the elderly, and once the disease manifests, the mortality and disability rates are relatively higher, further exacerbating the disease burden. Additionally, certain age-related brain changes in the elderly also increase their susceptibility to IS (32).

The GBD 2021 study on neurological disease burden (5) emphasized that in regions with Middle SDI, the diagnostic and monitoring capabilities for cardiovascular diseases are stronger, facilitating the detection and recording of IS cases, thereby resulting in higher incidence and prevalence data. Concurrently, higher-income elderly populations often exhibit poor lifestyle habits and are exposed to risk factors such as environmental pollution, thereby elevating their risk of IS. The situation is markedly different in low SDI regions. Although the actual incidence may be higher, poor medical conditions and a lack of necessary diagnostic equipment and professional staff make accurate reporting difficult, resulting in seemingly lower data. Residents in these regions often face basic health issues such as malnutrition and infectious diseases, which obscure the true incidence of IS. Due to economic constraints, residents in impoverished areas may not receive timely check-ups, diagnoses, or treatments, with many patients delaying diagnosis because of an inability to afford medical costs. Marshall et al. (33) demonstrated that SES has a significant impact on stroke risk and outcomes. Low SES is often associated with higher mortality rates, more severe symptoms, and diminished functional status. Low SDI regions lack sufficient medical resources and emergency response capabilities, making it difficult for elderly patients to be transferred to hospitals in a timely manner, leading to delayed treatment and poor recovery outcomes. This results in long-term disabilities, reduced quality of life, and higher mortality and DALY rates. In contrast, high SDI regions have better medical resource allocation, advanced technology, and comprehensive pre-hospital emergency care—postoperative rehabilitation system, which enable rapid treatment for acute patients and effective post-stroke rehabilitation, thereby reducing mortality and DALY rates.

In 2021, the ASIR was highest in East Asia, the ASPR was highest in Sub-Saharan Africa, and the ASMR and ASDR were highest in Eastern Europe. In contrast, the ASIR was lowest in the Andes region of Latin America, the ASPR was lowest in South Asia, and the age-standardized mortality and DALYs were lowest in Australasia. Zhang et al.’s research (34) indicates that East Asia, experiencing rapid economic development in countries such as China and Japan, has undergone a shift toward more Westernized lifestyles, including dietary habits and reduced physical activity, increasing the risk of IS. Furthermore, the region’s accelerated population aging has led to a larger underlying elderly population, further exacerbating the risk of IS. Camlet et al. (35) found that in Sub-Saharan Africa, some countries have relatively low economic development and scarce medical resources, which significantly affect the diagnosis, treatment, and management of IS. Additionally, the region faces multiple risk factors, such as HIV infections and indoor air pollution, which can have more severe effects on elderly populations, further exacerbating the disease burden. Dokova and Feigin (36) pointed out that Eastern Europe faces many challenges during socioeconomic restructuring, and inefficient medical resource allocation and utilization have adversely affected the treatment outcomes and disease management of IS in the elderly. In some Eastern European countries, such as those in the Balkans, acute stroke care quality may be lower, which adversely impacts patient outcomes and increases the disease burden.

Our study found that the most significant downward trends in the disease burden of IS in the elderly were observed in some high SDI regions, as well as in Western Europe, Eastern Europe, Central Europe, Australasia, and Southern Latin America, consistent with the findings of Fan Jiahui et al. The study suggests that in countries with similar SDI levels, the burden of IS varies, likely due to differences in healthcare systems and prevention efforts. Countries with stronger healthcare infrastructure and comprehensive preventive strategies tend to experience a lower disease burden (37). This study concludes that although the overall trend is declining, differences in disease burden changes exist across regions and populations. Governments should identify key regions and populations for disease prevention and control and formulate corresponding public health strategies.

The global aging process will continue to accelerate over the next decade, but our BAPC prediction results indicate that the disease burden of IS among elderly individuals will persistently decline globally by 2030. However, the number of elderly IS patients will still increase, with different age groups showing varying downward trends, which are more pronounced in the older age cohorts. Other studies suggest that the most effective and cost-saving strategies to reduce IS-related mortality in the elderly are those that specifically target risk factors. Therefore, controlling the additional mortality burden caused by high fasting blood glucose and high BMI should be prioritized globally (1). For males, the focus should be on strengthening smoking and alcohol cessation campaigns and interventions, while for older females, attention should be directed toward postmenopausal hormone replacement therapy and cardiovascular disease risk factor management (38). In terms of healthcare systems, further improvement in chronic disease management is needed, along with the global implementation of universal primary prevention strategies, in high-SDI regions, efforts should prioritize implementing AI-driven risk stratification systems for targeted prevention (39), while community health programs expand evidence-based exercise interventions using standardized testing tools (Graded Exercise Testing, Metabolic Equivalents) to develop individualized regimens for older stroke survivors (40). In contrast, low-SDI regions require a distinct, targeted approach. Specifically, policies should concentrate on enhancing public health infrastructure—such as establishing accessible acute stroke care facilities (41)—and on launching community-level education and screening programs tailored to the local epidemiological context (42). These measures aim to address the unique challenges of resource-limited settings and to mitigate the long-term burden of post-stroke disability among the elderly.

Future research should build upon the successes achieved in high SDI regions in reducing disease burden, such as low-cost stroke screening or rehabilitation services and optimized acute-phase treatment. These successful models should be adaptively trialed and validated in low-middle SDI regions, providing a viable pathway to narrowing global health disparities (43). By leveraging machine learning algorithms and mHealth technologies, a comprehensive investigation into the precise prediction and early intervention strategies for IS risk in the elderly could open new avenues for enhancing elderly health (44). The proactive establishment of an international, multicenter collaborative research platform—coupled with the dynamic optimization of resource allocation — will facilitate the integration of regional experiences and promote global public health equity. In view of the increasingly prominent challenges posed by ultra-aging populations, it is of great importance to conduct dynamic analyses of the pathophysiological characteristics of IS in the elderly. Special attention should be paid to accelerated vascular aging, the coexistence of multiple diseases, and the unique needs of patients in palliative care and functional rehabilitation (45). On this basis, constructing an integrated prevention and treatment model based on age stratification that seamlessly connects disease prevention, acute treatment, and rehabilitation management is imperative (46, 47). Moreover, beyond traditional risk factors such as hypertension and smoking, it is essential to consider the impacts of environmental exposures—such as fine particulate matter (48) and extreme climatic conditions (49)—and psychosocial stressors including loneliness and care dependency on the risk of IS among the elderly (50). Quantifying the dose–response relationships of these factors through multicenter cohort studies and environmental big data analyses will provide robust evidence for the development of precise prevention and treatment strategies.

Our study has several limitations. First, the data primarily originate from the GBD 2021 database. Although the database employs a standardized approach for data collection and indicator assessment, some regions, such as sub-Saharan Africa and Latin America, suffer from unreliable mortality information systems and population registries, which can affect the quality and accuracy of the data. This may result in biases in the assessment of IS in these regions and prevent the accurate reflection of the true disease burden. Second, when assessing disease burden, certain factors, such as differences in stroke subtypes, patients’ functional status, and regional variations in healthcare quality, may not have been fully considered. Different subtypes of IS in elderly patients may exhibit variations in pathogenesis, treatment response, and prognosis, but this study did not distinguish them in detail, which could compromise the accuracy of disease burden assessment. Additionally, healthcare quality directly impacts treatment outcomes and prognosis, but due to data limitations, this study could not comprehensively assess the effect of healthcare quality across regions. Third, this study did not account for the potential effects of sudden events (such as the global COVID-19 pandemic or natural disasters) on the disease burden of IS. These events may alter population health behaviors, healthcare resource allocation, and disease transmission patterns, thereby affecting disease incidence and progression trends.

## Data Availability

Publicly available datasets were analyzed in this study. This data can be found at: https://vizhub.healthdata.org/gbd-results.

## Footnotes

^1^ https://vizhub.healthdata.org/gbd-results/
