# Supplementary figures and images for: Global, regional, and national burden of ischemic stroke in older adults (≥60 years) from 1990 to 2021 and projections to 2030
Source: Front Neurol. 2025 May 8;16:1567609. doi: 10.3389/fneur.2025.1567609 (PMC12094992; doi:10.3389/fneur.2025.1567609)

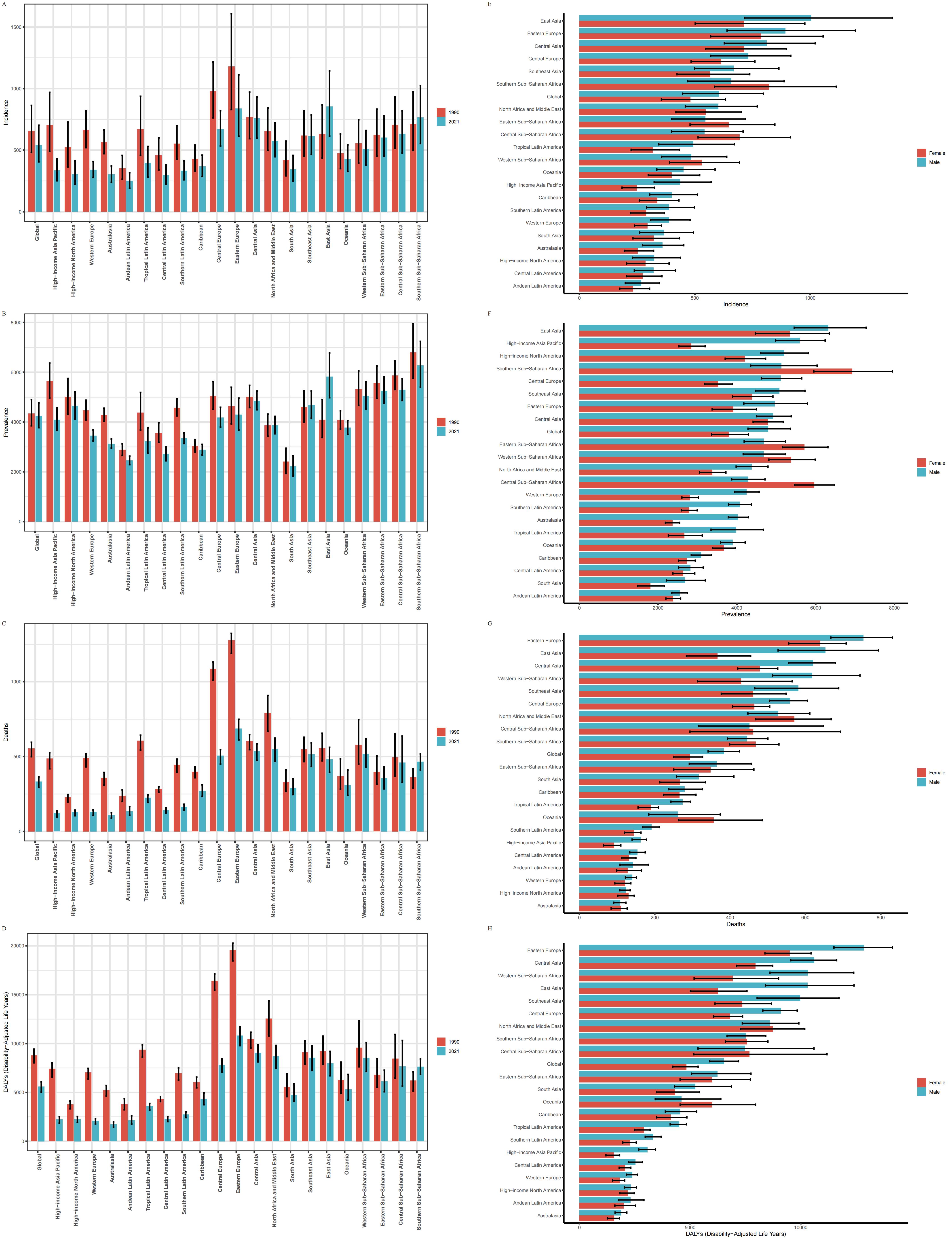

Supplement: Supplementary file 1 [file Image_1.jpeg]

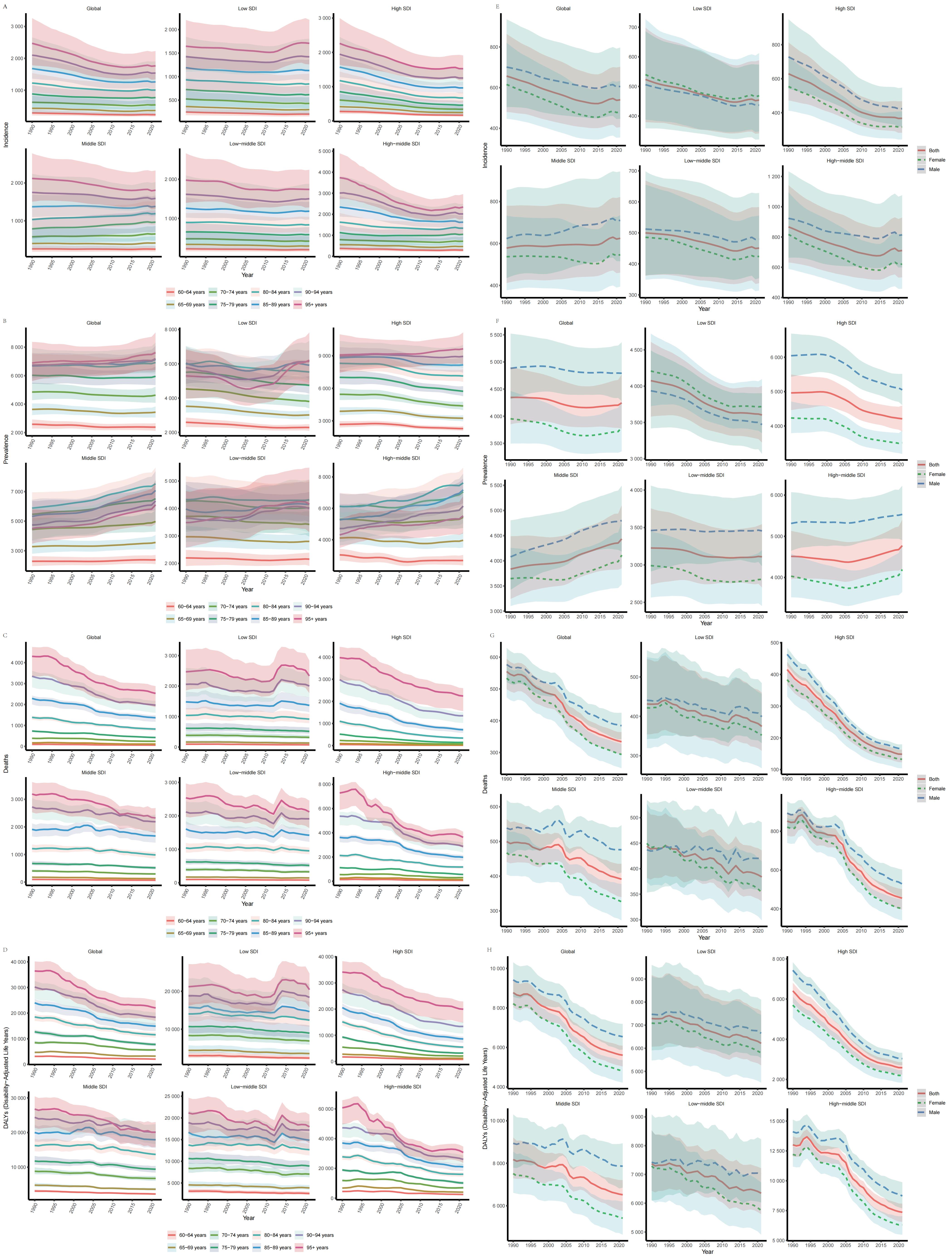

Supplement: Supplementary file 2 [file Image_2.jpeg]

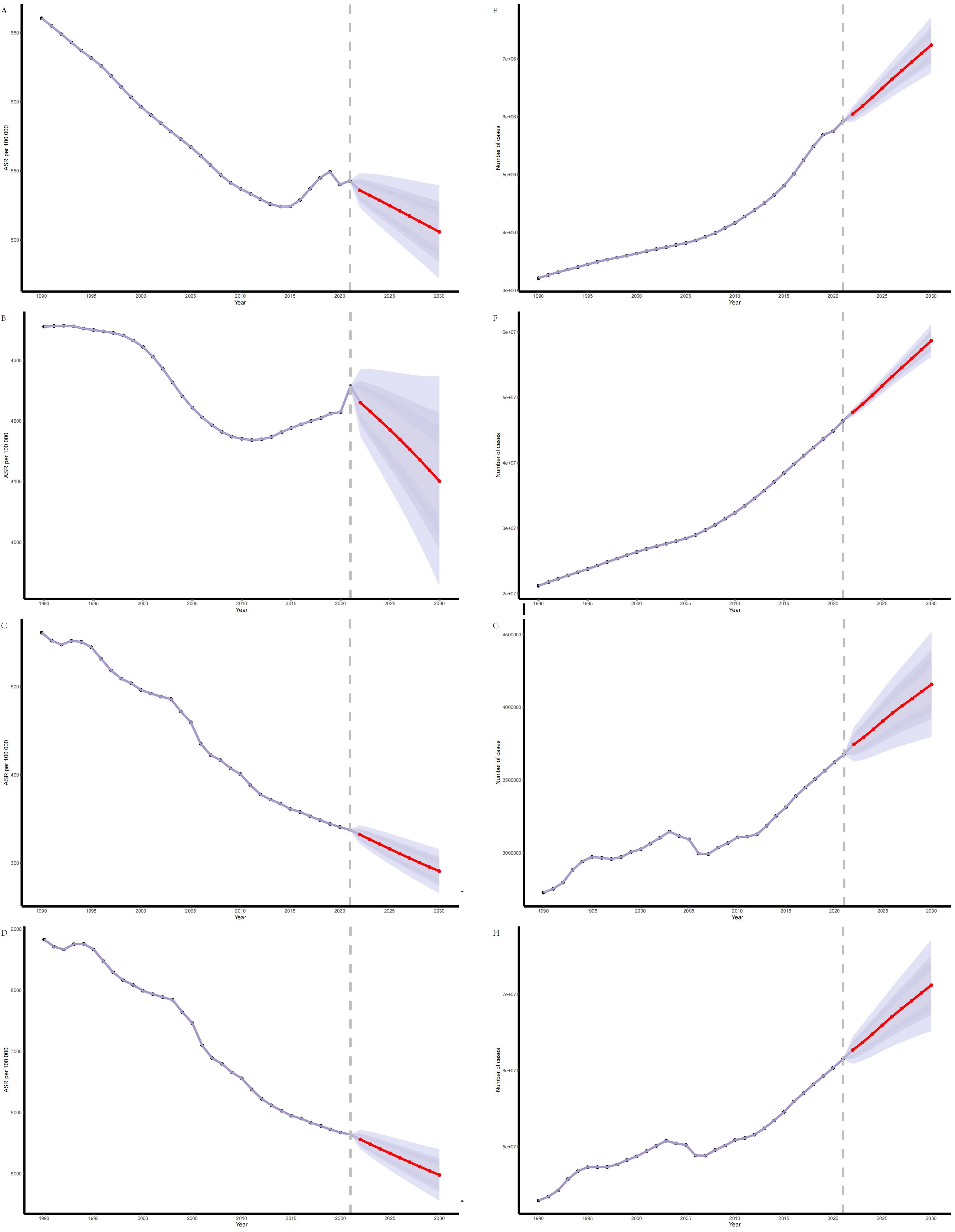

Supplement: Supplementary file 3 [file Image_3.jpeg]
